# Graphene-Supported Spinel CuFe_2_O_4_ Composites: Novel Adsorbents for Arsenic Removal in Aqueous Media

**DOI:** 10.3390/s17061292

**Published:** 2017-06-05

**Authors:** Duong Duc La, Tuan Anh Nguyen, Lathe A. Jones, Sheshanath V. Bhosale

**Affiliations:** 1 School of Science, RMIT University, GPO Box 2476, Melbourne, VIC 3001, Australia; duc.duong.la@gmail.com (D.D.L.); lathe.jones@rmit.edu.au (L.A.J.); 2 Applied Nanomaterial Laboratory, ANTECH, Hanoi 100000, Vietnam; Tuananhnguyendhb@gmail.com; 3 Centre for Advanced Materials and Industrial Chemistry (CAMIC), School of Science, RMIT University, GPO Box 2476, Melbourne, VIC 3001, Australia

**Keywords:** graphene-supported CuFe_2_O_4_ composite, graphene nanoplates, spinel CuFe_2_O_4_, arsenic removal, graphene-oxide hybrid material

## Abstract

A graphene nanoplate-supported spinel CuFe_2_O_4_ composite (GNPs/CuFe_2_O_4_) was successfully synthesized by using a facile thermal decomposition route. Scanning electron microscopy (SEM), high resolution transmission electron microscopy (HRTEM), Electron Dispersive Spectroscopy (EDS), X-ray diffraction (XRD) and X-ray Photoelectron Spectroscopy (XPS) were employed to characterize the prepared composite. The arsenic adsorption behavior of the GNPs/CuFe_2_O_4_ composite was investigated by carrying out batch experiments. Both the Langmuir and Freundlich models were employed to describe the adsorption isotherm, where the sorption kinetics of arsenic adsorption by the composite were found to be pseudo-second order. The selectivity of the adsorbent toward arsenic over common metal ions in water was also demonstrated. Furthermore, the reusability and regeneration of the adsorbent were investigated by an assembled column filter test. The GNPs/CuFe_2_O_4_ composite exhibited significant, fast adsorption of arsenic over a wide range of solution pHs with exceptional durability, selectivity, and recyclability, which could make this composite a very promising candidate for effective removal of arsenic from aqueous solution. The highly sensitive adsorption of the material toward arsenic could be potentially employed for arsenic sensing.

## 1. Introduction

Arsenic is highly toxic in the +3 and +5 oxidation state, and is widely present in the environment through leaching from soils, mining activities, fertilizers, industrial wastes, biological activity, and naturally occurring As containing minerals [1,2]. Long-term ingestion and drinking of arsenic contaminated food or water are linked to kidney, skin and lung cancers [3,4,5,6]. Therefore, it is of continued importance to remove arsenic from contaminated water, and to provide safe drinking water below the maximum concentration recommended by WHO (<10 ppb). Many approaches have been used for arsenic removal from contaminated water, including adsorption, ion exchange, chemical treatment, reverse osmosis, electrochemical treatment, membrane filtration, and co-precipitation [7,8,9,10]. However, due to its simplicity, low cost and high efficiency, adsorption is widely employed and studied as a promising technology for effectively removal of arsenic from contaminated water. The simplicity of these materials is especially important when it is recognized that As contamination is common in the developing world, where treatment processes must be convenient and affordable.

Many adsorbents based on agriculture and industrial waste, surfactants, carbon-base materials, polymers and metal oxides have been employed for arsenic adsorption [11,12]. Among these, metal and metal oxides such as TiO_2_ [13,14,15], nano zero-valent iron [16,17], Fe_2_O_3_ [3,18,19], Fe_3_O_4_ [20], CeO_2_ [21], CuO [22,23], CaO [24] and ZrO_2_ [25,26] have been extensively studied for arsenic treatment in aqueous solution because of their high affinity to arsenic species, low cost, and the tunability of adsorption capacity [12,27]. Recently, considerable attention has been focused on the development of adsorbent composites containing two or more metals as metal oxides, to maximize arsenic adsorption. For instance, Zhang and co-workers synthesized a nanostructured Fe-Cu binary oxide with high adsorption capacity for arsenic [28].

Fe-Mn binary oxides were also successfully fabricated by Shan et al. with a high adsorption capacity toward arsenic [29]. In another report, Yu et al. presented Fe–Ti binary oxide magnetic nanoparticles which combined the photocatalytic oxidation property of TiO_2_ with the high adsorption capacity and magnetic properties of γ-Fe_2_O_3_, for arsenic treatment [30]. Basu et al. found that Fe(III)-Al(III) mixed oxides and Fe(III)-Ce(IV) oxides have a high adsorption capacity toward arsenic [31,32].

Graphene, a two-dimensional (2D) material, has been attracting significant interest in the past decade, due to its exceptional chemical and physical properties which can be applied to many different areas including, but not limited to, electronic devices, energy storage and conversion, sensors, adsorption, and composites [33,34,35,36,37,38]. Most recently, graphene has gained tremendous interest as a supporting material for enhancement of adsorption properties of adsorbents, due to its large surface area, high conductivity, ionic mobility, and superior mechanical flexibility. For example, Ganesh et al. reported a smart magnetic graphene that removed heavy metals from drinking water [39]. A hybrid of monolithic Fe_2_O_3_/graphene was also fabricated, and showed favorable properties for arsenic removal [40]. Reduced graphene oxide-supported mesoporous Fe_2_O_3_/TiO_2_ nanoparticles synthesized by a sol-gel route showed high adsorption towards arsenic [41]. Kumar et al. synthesized single-layer graphene oxide with manganese ferrite magnetic nanoparticles for efficient removal of arsenic from contaminated water [42].

In our previous work, we successfully fabricated a graphene nanoplates (GNPs) -supported Fe-Mg binary oxide composite by a simple hydrothermal method. This adsorbent showed a very high adsorption capacity toward arsenic [43]. In continuation of our efforts to this end, herein we report a simple one-pot hydrothermal method to prepare a graphene nanoplates-supported spinel CuFe_2_O_4_ (GNPs/CuFe_2_O_4_) composite. The optimized Cu:Fe molar ratio to fabricate the spinel CuFe_2_O_4_ for arsenic adsorption was adopted from Zhang’s work, which is 1:2 [28]. TEM, SEM, EDS, TGA, XPS and XRD were used to characterize the prepared composite. The arsenic adsorption capacity of the material was carefully studied. The effects of parameters including graphene loading, initial arsenic concentration, adsorption time and solution pH on arsenic adsorption, selectivity, and recyclability were investigated through batch experiments and a column test.

## 2. Materials and Methods

### 2.1. Materials

Graphene nanoplates (GNPs) were obtained from VNgraphene. Dry acetone, ethanol, sodium hydroxide (NaOH), posstasium hydroxide (KOH), sodium persulfate (Na_2_S_2_O_8_), As_2_O_5_, anhydrous CuCl_2_ and FeCl_3_ were purchased from Ajax Finechem. All chemicals were used as received.

### 2.2. Synthesis of GNPs/CuFe_2_O_4_ Composite

GNPs/CuFe_2_O_4_ composites were fabricated by a simple one-pot hydrothermal strategy. Firstly, CuCl_2_ and FeCl_3_ with various Cu:Fe molar ratios of 1:2 were dissolved in 50 mL of ethanol. Then graphene nanoplates with different loadings were dispersed in the mixture solution by sonication for 10 min, and then stirred for 1 h. Subsequently, a 2 M NaOH solution was added dropwise to the solution under vigorous stirring until a pH of ~8–9 was reached. After 1 h of further stirring, the reaction solution was transferred and sealed in a Teflon-lined autoclave, and placed in an oven pre-heated to 150 °C, for 2 h. Then the solution was cooled to room temperature, and the precipitate was filtered and washed three times each with ethanol and distilled water. The sample was dried overnight at a temperature of 60 °C in air to obtain the GNPs/CuFe_2_O_4_ composites.

### 2.3. Characterization

The morphology and mapping elemental composition of samples were studied by an EDS-equipped (Oxford Instruments plc, Abingdon, Oxfordshire, UK) scanning electron microscope using an FEI Nova NanoSEM (Hillsboro, AL, USA) operating under high vacuum with an accelerating voltage of 30 keV and an Everhart Thornley Detector (ETD). HRTEM images were obtained on a JEOL 2010 TEM instrument operated at an accelerating voltage of 100 kV. A BrukerAXS D8 Discover instrument with a general area detector diffraction system (GADDS) using a Cu Kα source was utilized to obtain XRD patterns. X-ray photoelectron spectra (XPS) were obtained on a K-Alpha XPS instrument using monochromated aluminum as the X-ray source. The C 1s, Fe 2p, Cu 2p, As 3d and O 1s core level spectra were recorded with an overall resolution of 0.1 eV. The core level spectra were background corrected using the Shirley algorithm, and chemically distinct species were resolved using a nonlinear least square fitting procedure.

### 2.4. Adsorption Studies

A stock solution of 1000 ppm As(V) was prepared by dissolving As_2_O_5_ in water. Arsenic concentrations were determined using an Agilent 4200 microwave plasma-atomic emission spectrometer (MP-AES). All samples were analyzed within 24 h of filtration.

#### 2.4.1. Effect of GNPs Loading on Arsenic Sorption

Adsorption experiments were carried out in closed glass vessels. Typically, 24 mg of each adsorbent prepared from different GNP loadings were added into glass vessels containing 50 mL of 10 mg/L arsenic solution at a pH of 4. The solution was kept shaking at 200 rpm at room temperature for 24 h. Then, all samples were filtered by vacuum filtration to remove the adsorbent, and the concentration of arsenic in the residual solutions was analyzed.

#### 2.4.2. Adsorption Isotherm

A total of 10 mg of optimally fabricated adsorbent was added to 50 mL of As(V) solution with initial concentrations ranging from 5 to 90 mg/L in glass vessels. The adsorption was carried out at a solution pH of 4 at room temperature, shaking at a speed of 200 rpm for 24 h. The mixtures were then filtered by vacuum filter and analyzed for residual arsenic by microwave plasma—atomic emission spectrometry (MP-AES).

#### 2.4.3. Adsorption Kinetics

In a typical experiment, 40 mg of GNPs/CuFe_2_O_4_ composite was mixed with 200 mL of 40 mg/L arsenic in a glass vessel. The mixed solution was shaken on an orbital shaker at a speed of 200 rpm at room temperature and solution pH of 4. At certain time intervals, 10 mL of the mixture was taken, filtered by vacuum filter and analyzed for arsenic.

#### 2.4.4. Effect of Solution pH

10 mg of GNPs/CuFe_2_O_4_ composite was added to 50 mL of 10 mg/L arsenic at various solution pH values ranging from 4 to 11 (the pH values were adjusted by dilute HCl and NaOH solutions). The suspensions were shaken at a speed of 200 rpm at room temperature for 24 h. Then, all samples were filtered by vacuum filter and residual arsenic concentrations were determined.

#### 2.4.5. Selectivity Test

10 mg of GNPs/ CuFe_2_O_4_ adsorbent was added to 50 mL of a solution containing 3 mg/L of each ion such as As(V), Na^+^, K^+^, Ca^2+^ and Mg^2+^. The mixed solution was shaken on an orbital shaker with a speed of 200 rpm at room temperature and solution pH of 7 for 12 h. The mixtures were then filtered by vacuum filter and analyzed for residual ions by MP-AES.

#### 2.4.6. Recyclability Test

The reusability of the prepared adsorbent was studied by a column test. The GNPs/CuFe_2_O_4_ oxide composite was assembled as a part of a filter column. Other parts included a glass tube with both ends wrapped with a few layers of filter papers and cotton. The filter column was regenerated by washing several times with 2 M NaOH after each arsenic adsorption cycle before implementing the next experiment. 

## 3. Results and Discussion

**Figure 1:**
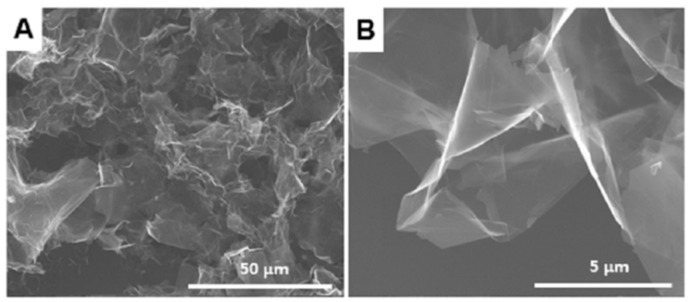
Low (**A**) and high (**B**) scanning electron microscopy (SEM) images of graphene nanoplates.

The morphology of the obtained graphene nanoplates (GNPs) was studied by SEM (Figure 1). It can be clearly seen in Figure 1a,b that the GNPs had a crumpled, wrinkled morphology with a diameter of tens of microns and a thickness of <20 nm [44].

**Figure 2:**
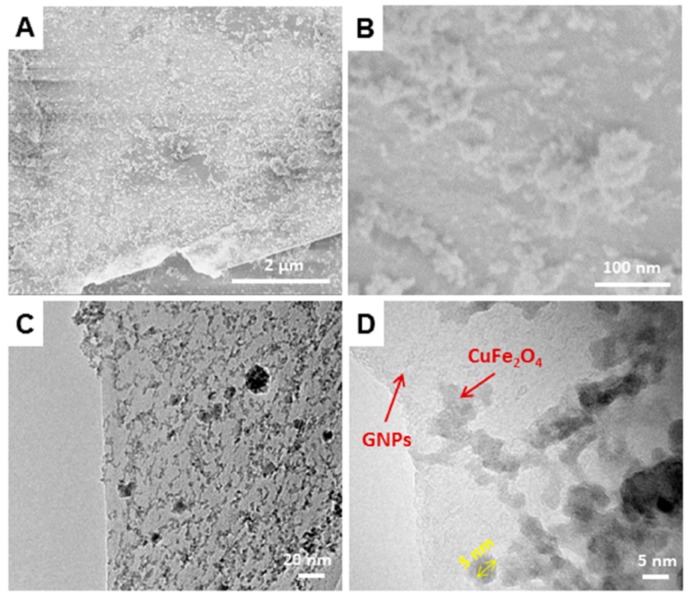
(**A**) and (**B**) SEM images and (**C**) and (**D**) transmission electron microscopy (TEM) images of the GNPs/CuFe_2_O_4_ composite.

**Figure 3:**
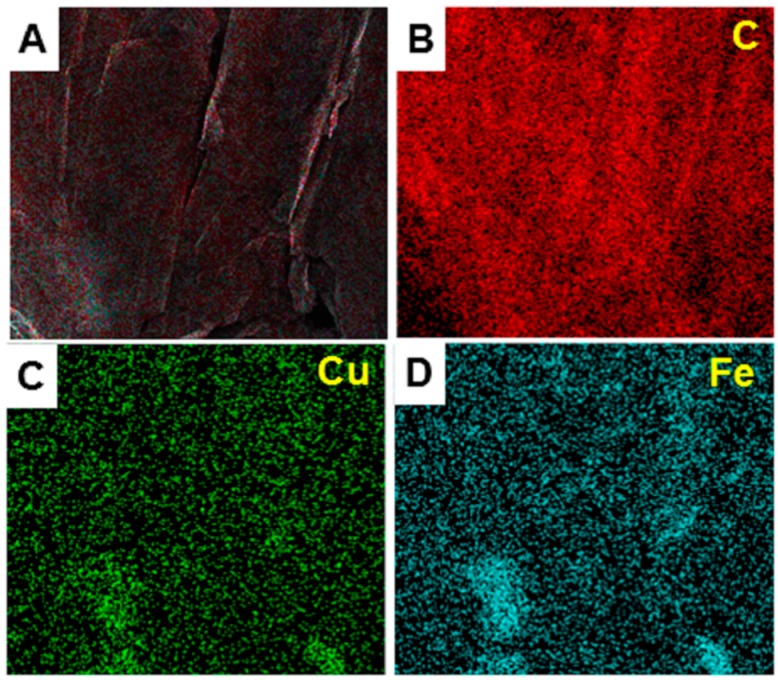
Electron dispersive spectroscopy mappping of the GNPs/CuFe_2_O_4_ composite.

It is believed that the Fe^3+^ and Cu^2+^ ions firstly physically adsorbed on the GNPs, and then these ions reacted under the hydrothermal conditions to form Fe-Cu binary oxides on the GNPs. The morphology of the as-prepared GNPs/Fe-Cu binary oxides material was investigated by SEM and HRTEM studies. Figure 2a,b and Figure S1 show low and high resolution of SEM images of the composites on a silicon wafer. The SEM images confirmed that the Fe-Cu binary oxides were uniformly dispersed on the surface of the GNPs. The low resolution HRTEM image shown in Figure 2C and Figure S2, also confirmed a good distribution of oxides on the GNPs. When the composite was viewed at the high resolution of HRTEM (Figure 2D), it can be clearly seen that Fe-Cu binary oxides were well-separated, with particle sizes of approximately 5 nm in diameter. The uniform distribution of Fe-Cu binary oxides on the GNPs was further confirmed by EDS mapping (Figure 3). The distribution of Cu and Fe elements on the surface of graphene (elemental C) was uniform and homogeneous.

**Figure 4:**
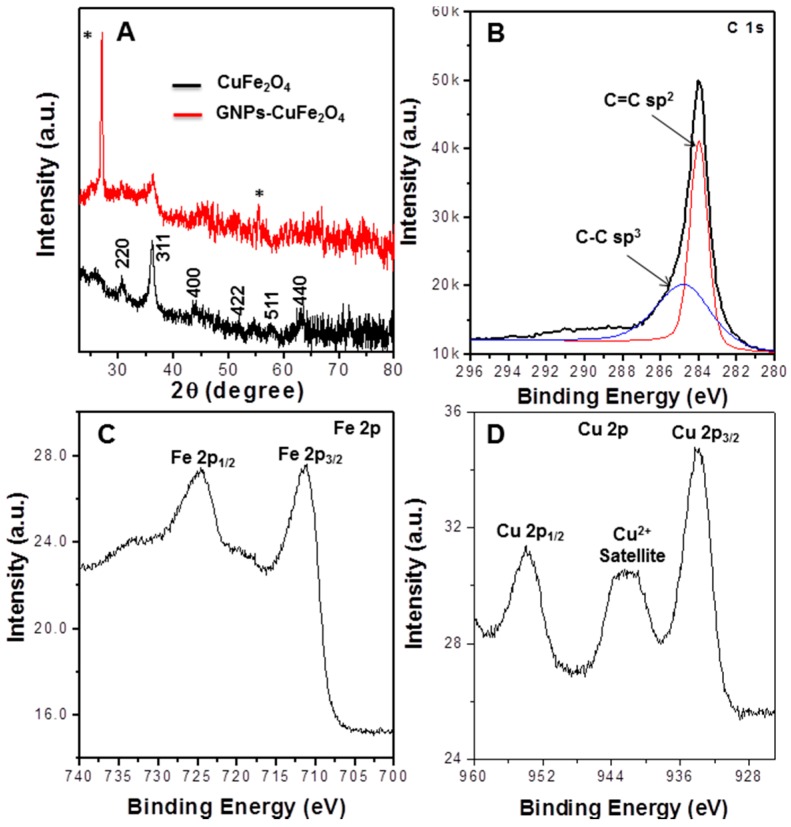
(**A**) X-ray diffraction (XRD) patterns of pure spinel CuFe_2_O_4_ and GNPs/CuFe_2_O_4_ composites; (**B**–**D**) core level X-ray Photoelectron Spectroscopy pectra of C 1s, Fe 2p and Cu 2p, respectively, obtained from the GNPs/CuFe_2_O_4_ composite.

The EDS study also confirmed that the atomic ratio of Cu:Fe:O is approximately 1:2:4, which was consistent with the theoretical formula of the spinel CuFe_2_O_4_, or a mixture of CuO and Fe_2_O_3_ oxides formula of obtained binary oxides. In order to further confirm the formation of metal oxides, XRD diffraction patterns were obtained. Figure 4A shows the XRD pattern of the pure CuFe_2_O_4_ and GNPs/Fe-Cu binary oxides composite. In the XRD spectrum of the pure CuFe_2_O_4_, the diffraction peaks at 2θ = 30.56, 36, 43.7, 51.8, 56.6, and 63.1° could be indexed to the (220), (311), (400), (422), (511), and (440) planes of cubic spinel CuFe_2_O_4_ (PDF 06-0545) [45]. When incorporated onto the GNPs surface, the metal oxides mainly formed the cubic spinel structure of CuFe_2_O_4_, as the main diffraction peaks matched with the pure CuFe_2_O_4_. The peaks with asterisks were attributed to the crystallites of the supporting graphene nanoplates [44,46]. 

The core level XPS spectra of C 1s, Fe 2p and Cu 2p were obtained to probe the chemical environment and oxidation states of C, Fe and Cu in the GNPs/CuFe_2_O_4_ composite, as exhibited in Figure 4B–D. The deconvoluted core level of C 1s (Figure 4B) revealed two major peaks at 284.1 and 284.8 eV corresponding to graphenic carbon with C=C (sp2) and C-C (sp3) bonds [47], respectively. In the Figure 4C, the Fe core level XPS spectrum had two dominant peaks at 711.18 and 724.28 eV with small satellite, which was consistent with the Fe 2p3/2 and Fe 2p1/2 of the Fe^3+^ state in the spin-orbit of CuFe_2_O_4_, respectively [48]. Figure 4D showed the binding energy of core level Cu 2p. The fitting revealed peaks at around 933.78 and 953.78 eV with a broad satellite at around 942 eV, which corresponded to the Cu2 2p_3/2_ and 2p_1/2_, respectively, of Cu^2+^ in the spinel CuFe_2_O_4_ [48]. All of these results further confirmed the formation of CuFe_2_O_4_ on the GNPs.

**Figure 5:**
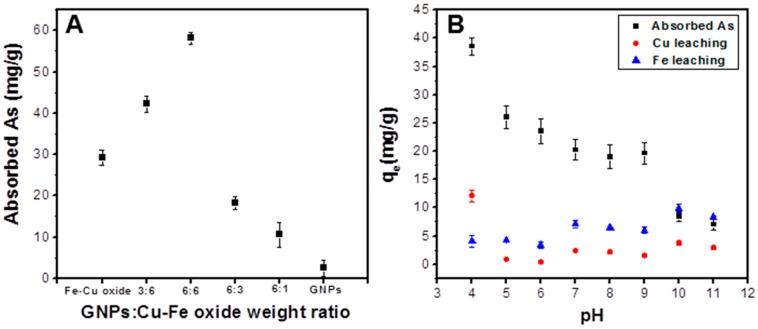
Effect of graphene nanoplates (GNPs) loading (**A**) and solution pH (**B**) on As (V) adsorption by GNPs/CuFe_2_O_4_ composites.

It is of note that the As(V) sorption by the Fe-Cu binary oxide reaches a maximum when the molar ratio of Cu:Fe is 1:2 [28]. Hence, in this study, we have chosen this molar ratio as an optimal condition when preparing the spinel CuFe_2_O_4_. We then investigated the effect of graphene loading on the arsenic sorption capacity by the GNPs/CuFe_2_O_4_ composite with an initial As(V) concentration of 24 mg/L, adsorbent dose = 200 mg/L, pH = 4, at room temperature (Figure 5A). It was seen from Figure 5 that the As(V) adsorption was enhanced along with an increase in the GNPs loading, and reached a maximal sorption capacity of about 58 mg/g at the GNPs:CuFe_2_O_4_ weight ratio of 1:1 (6:6 in the figure). However, the sorption capacity dramatically dropped as GNPs loading increased above 1:1, and without CuFe_2_O_4_, the As(V) sorption capacity by pure GNPs was only 2.38 mg/g. These results demonstrated a significant improvement in As(V) adsorption with the incorporation of GNPs with CuFe_2_O_4_. 

Figure 5B shows the effect of pH on As(V) adsorption by the GNPs/CuFe_2_O_4_ composite at an initial As(V) concentration of 10 mg/L, and adsorbent dose = 200 mg/L at room temperature. It is obvious that the sorption capacity of As(V) strongly depended on the solution pH. Arsenic adsorption occurred strongly in acidic conditions with a maximum capacity of 39 mg/g at pH = 4. When the solution pH increased, the adsorption significantly decreased. The change in As(V) adsorption capacity was negligible in the solution pH range of 5 to 9 before declining greatly when the solution pH further increased. This phenomenon may be ascribed to the dependence of adsorption of strong acid anions by metal oxides and hydroxides oxide in solution pH [28]. The p*K_a_*1, p*K_a_*2, p*K_a_*3 of As(V) are 2.1, 6.7, 11.2, which is present in a negative ionic form under most pH conditions. Since the electrostatic attraction is the main force, which is responsible for the adsorption of As(V) on graphene-metal oxide composites [49,50], the change of electrostatic force between As(V) and the GNPs/CuFe_2_O_4_ composite may explain the effect of pH on As(V) adsorption. At a low pH, the GNPs/CuFe_2_O_4_ adsorbent has a net positive charge due to protonation of –OH groups in the spinel CuFe_2_O_4_. As a result, they attract the negatively charged As(V) ions (AsO_4_^3−^), which leads to the greater adsorption. When the pH increases, the positive charge decreases, resulting in a decrease of As(V) adsorption.

The leaching of Fe and Cu in the GNPs/CuFe_2_O_4_ composite at different pH values was also recorded, as shown in Figure 5B. The release of Fe and Cu was low compared to As(V) adsorption, which indicated that the GNPs/CuFe_2_O_4_ composite was a stable and effective adsorbent for arsenic.

The adsorption isotherm was obtained in order to assess the arsenic adsorption and determine the maximum As(V) adsorption capacity by the GNPs/CuFe_2_O_4_ composite. The amount of arsenic adsorbed on the composite at equilibrium (*q_e_*) was calculated from different concentrations of arsenic with the following equation:

(1) $$q_{e}=\frac{\left(C_{0}-C_{e}\right)\times V}{m}$$

where *C*_0_ (mg/L) is the initial concentration, *C_e_* (mg/L) is the equilibrium concentration, *V* (L) is the solution volume, and *m* (g) is the mass of the GNPs/CuFe_2_O_4_ adsorbent. 

**Figure 6:**
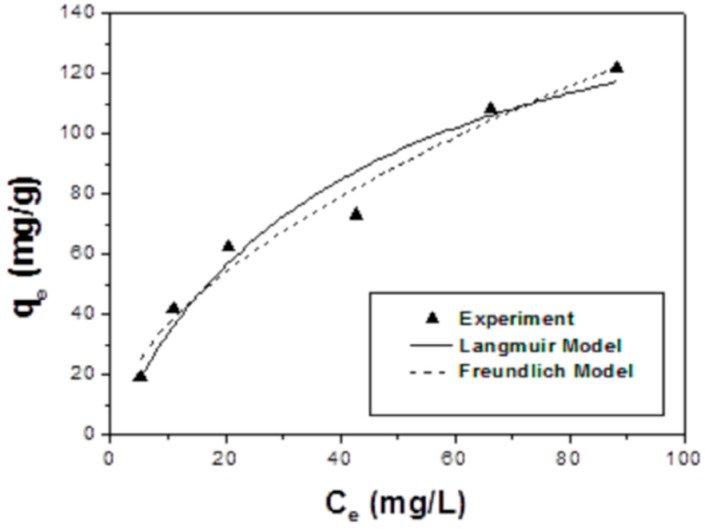
Adsorption isotherm for As(V) by the GNPs/CuFe_2_O_4_ composite.

Figure 6 shows the arsenic adsorption capacity by the composite at equilibrium with various As(V) concentrations in the range of 5–90 mg/L, at an adsorbent dose of 200 mg/L, pH 4 under room temperature. Both adsorption isotherms for the Langmuir and Freundlich models were used to fit the data as expressed in Equations (2) and (3), respectively:

(2) $$q_{e}=\frac{q_{max}K_{L}C_{e}}{1+K_{L}C_{e}}$$

(3) $$q_{e}=K_{F}C_{e}^{n}$$

where *q_e_* is the amount of arsenic adsorbed on the solid phase at equilibrium (mg/g), *q_max_* (mg/g) is the maximum arsenic adsorption capacity per unit weight of adsorbent, *C_e_* is the equilibrium arsenic concentration (mg/L), *K_L_* is the equilibrium adsorption constant represented by the affinity of binding sites (L/mg), *K_F_* is the Freundlich constant, and *n* is the heterogeneity factor.

The obtained As(V) adsorption constants are presented in Table 1. The higher correlation coefficient (0.966) values of As(V) from the fitted Freundlich plots compared to that of Langmuir plots (0.95) suggested that the Freundlich model was more suitable for representing the adsorption behavior of As(V) by the GNPs/CuFe_2_O_4_ composite. The low calculated heterogeneity factor (n = 0.56 for As(V)) also suggests that the Freundlich was the more favorable model. These results indicate that As(V) was heterogeneously adsorbed on the composite surface, suggesting the simultaneous existence of graphene and iron-copper binary oxides in the solid phase. The maximum As(V) adsorption capacity by the GNPs/CuFe_2_O_4_ determined from the Langmuir model was 172.27 mg/g, which was a very effective adsorbent for the removal of arsenic. The maximum As(III) adsorption capacity by the GNPs/CuFe_2_O_4_ was also determined from the Langmuir model as 236.29 mg/g (Figure S3 and Table S1).

Table 2 compares the As(V) adsorption capacity between the GNPs/CuFe_2_O_4_ composite with other adsorbents from the literature. It can be seen from the table that the sorption capacity of the GNPs/CuFe_2_O_4_ composite was superior to most of the other adsorbents, which could make the GNPs/CuFe_2_O_4_ composite a practical adsorbent for arsenic removal.

**Figure 7:**
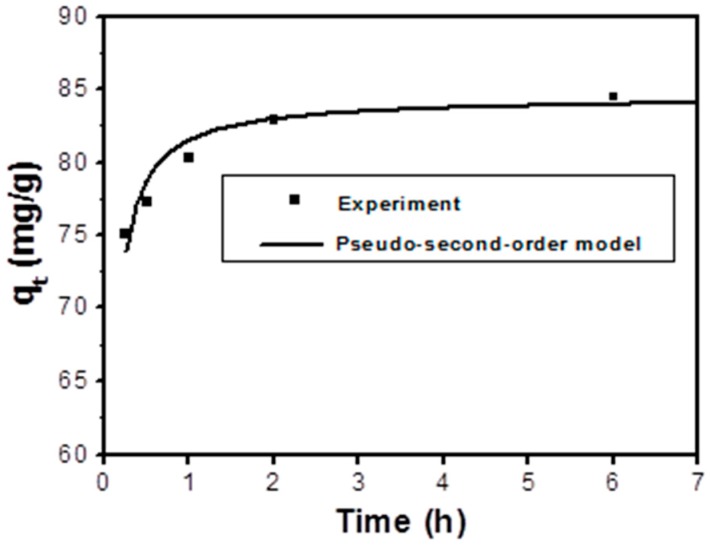
Adsorption kinetics of As(V) on the GNPs/CuFe_2_O_4_ composite.

In order to further understand the adsorption behavior of As(V) on the GNPs/CuFe_2_O_4_ surface, the adsorption kinetics of As(V) adsorption were obtained with an initial As(V) concentration of 40 mg/L (adsorbent dose of 200 mg/L, pH 4 and at room temperature), and sorption capacities were determined at different time intervals (Figure 7). The adsorption quickly reached equilibrium within 2 h. The pseudo-second-order model was applied to describe the kinetic data as expressed in Equation (4):

(4) $$q_{t}=\frac{Kq_{e}^{2}t}{1+Kq_{e}t}$$

where *q_t_* (mg/g) is the amount of arsenic adsorbed on the solid phase at time *t* (hr), *q_e_* (mg/g) is the amount of arsenic adsorbed on the solid phase at equilibrium, and *K* is the adsorption rate constant (g mg.h). According to the adsorption kinetic values listed in Table 3, the experimental data was well-fitted, with a correlation coefficient of 0.916. This result implies that the adsorption process may have occurred through chemical adsorption and/or electrostatic attraction, accompanied by electron exchange between the composite and arsenic [57]. The adsorption capacity at equilibrium of the composite with an initial As(V) concentration of 40 mg/L calculated from the pseudo-second-order model was 84.46 mg/g.

**Figure 8:**
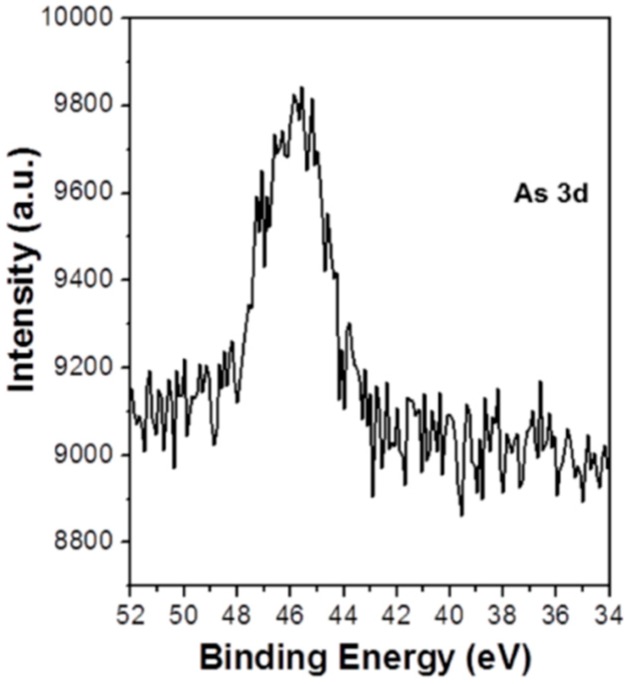
Core level XPS spectra of As 3d obtained from the GNPs/CuFe_2_O_4_ composite after adsorption.

The XPS As 3d core level spectrum was recorded to verify the presence and chemical state of arsenic on the surface of adsorbent (Figure 8). The appearance of As 3d XPS peak confirmed the presence of arsenic on the surface of the GNPs/CuFe_2_O_4_ composite. The core level XPS revealed one dominant peak at 45.5 eV, which was consistent with the binding energy of As(V) [28,58]. As a result, it was obvious that there was no change in oxidation state of As(V) during the sorption process. This further confirmed that arsenic was adsorbed onto the GNPs/CuFe_2_O_4_ surface by chemical adsorption and/or an electrostatic attraction mechanism.

**Figure 9:**
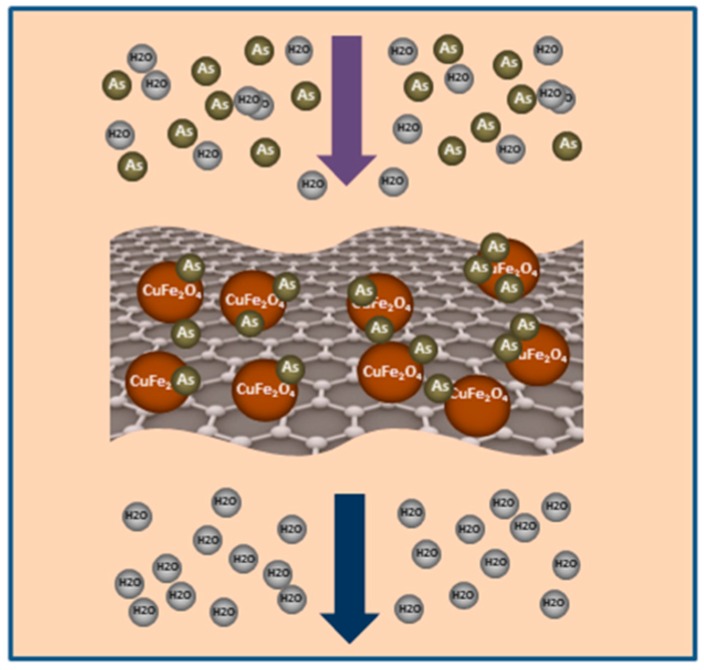
Possible adsorption mechanism of GNPs/Cu_2_FeO_4_ toward arsenic.

Graphene with a large surface area, high conductivity, ionic mobility and superior mechanical flexibility, can be an excellent supporting material for the enhancement of adsorption properties of adsorbents. In this case, GNPs/CuFe_2_O_4_ adsorbent showed enhanced adsorption capacity in comparison with free standing CuFe_2_O_4_ (*q*_max_ = 82.7 mg/g). Based on well-documented understanding and from the discussion above, we proposed a possible adsorption of arsenic by GNPs/CuFe_2_O_4_ (Figure 9). When adding adsorbent into arsenic-containing solution, arsenic is adsorbed on the CuFe_2_O_4_ surface by chemical adsorption and/or electrostatic attraction. The presence of graphene increases the surface area of adsorbent, and as a consequence increases the absorption sites for arsenic.

**Figure 10:**
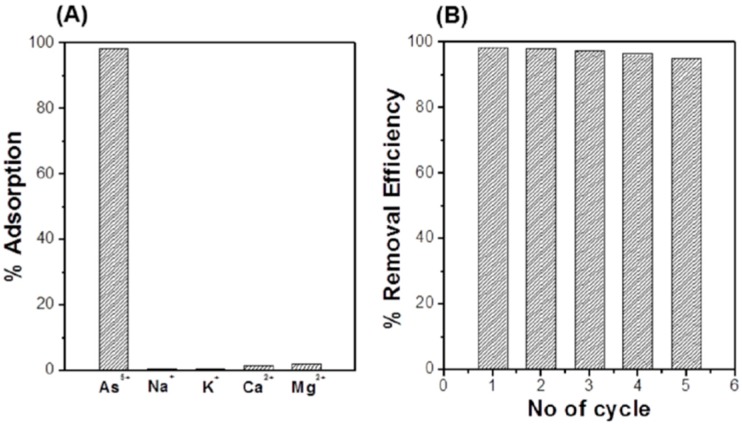
Selectivity (**A**) and recyclability (**B**) of the GNPs/CuFe_2_O_4_ composite for As(V) removal in a column test.

Figure 10A shows the selectivity of GNPs/CuFe_2_O_4_ adsorbent towards arsenic in the presence of common positive ions in drinking water such as Na^+^, K^+^, Ca^2+^ and Mg^2+^, with an initial concentration of As(V) and other ions at 3 mg/L. It can be seen that while more than 98% of arsenic was adsorbed, there was an insignificant amount of Na^+^, K^+^ and Ca^2+^ ions adsorbed on the GNPs/CuFe_2_O_4_ composite, indicating that this adsorbent can be effectively and selectively used for arsenic removal. The adsorption capacity of GNPs/CuFe_2_O_4_ composite toward arsenic was also higher than other heavy metals such as lead ions (Figure S4).

To evaluate recyclability, a filter column with a diameter of 2 cm and a height of 10 cm was assembled, as shown in Figure S5. The mass of the GNPs/CuFe_2_O_4_ composite used was 200 mg. The adsorption process was carried out with 10 mL of flow solution (pH 7) of 3 mg/L As(V). After adsorption, the filter column was washed with 20 mL of 2 M NaOH solution to regenerate the adsorbent before the next test cycle. The adsorption-regeneration process was repeated for five cycles. The result in Figure 10B shows an insignificant decrease of removal efficiency (less than 4%) after 5 cycles, suggesting that the GNPs/CuFe_2_O_4_ composite has high durability for arsenic removal.

## 4. Conclusions

A graphene-supported spinel CuFe_2_O_4_ composite was conveniently synthesized by co-precipitating graphene nanoplates with iron and copper ions in ethanol solution. The CuFe_2_O_4_ was crystallized and well-dispersed on the graphene surface. The prepared GNPs/CuFe_2_O composite showed fast, high adsorption capacity toward As(V), with a maximum adsorption capacity of 172.27 mg/g at pH 4, which is superior to the majority of reported adsorbents. This adsorbent showed excellent selectivity toward arsenic ions over common metal ions such as Na^+^, K^+^, Ca^2+^ and Mg^2+^. The arsenic adsorption by the GNPs/CuFe_2_O composite was very effective over a wide range of solution pHs. Moreover, the absorbent could be readily regenerated and recycled for arsenic removal. With these excellent results, it could be concluded that the GNPs/CuFe_2_O composite could be considered a promising candidate for practical arsenic removal from aqueous solution. Furthermore, the GNPs/CuFe_2_O composite can be potentially used as a sensor probe of arsenic, based on its high sensitive adsorption toward arsenic.

## Figures and Tables

**Table 1:** Langmuir and Freundlich isotherm parameters for As(V) adsorption on the GNPs/CuFe_2_O_4_ composite.

	Langmuir Model			Freundlich Model		
	*Q_m_* (mg/g)	*K_L_* (L/mg)	*R*^2^	*K_F_*	*n*	*R*^2^
As(V)	172.27	0.02	0.95	10.31	0.56	0.966

**Table 2:** Comparison of maximal arsenic adsorption capacity by various adsorbents.

Absorbates	pH	q_max_ (mg/g)	References
Mg_0.27_Fe_2.5_O_4_	7	83.2	[51]
Fe_3_O_4_-GO (MGO)	6.5	59.6	[49]
FeMnO_x_/RGO	7	22.22	[52]
CeO_2_-grahene composite	4	1.019	[53]
GO-ZrO(OH)_2_	5–11	84.89	[50]
nZVI/graphene	7	29	[54]
Magnetic graphene	4	3.26	[39]
Fe_3_O_4_/graphene/LDH	6	73.1	[39]
Magnetic-GO	4	38	[55]
Magnetic-rGO	4	12	[55]
MnFe_2_O_4_	3	94	[56]
CoFe_2_O_4_	3	74	[56]
CuFe_2_O_4_ binary oxide	7	82.7	[28]
GNPs/Fe-Mg Oxide	7	103.9	[43]
GNPs/CuFe_2_O_4_	4	172.7	This work

**Table 3:** Adsorption kinetics parameters for As(V) adsorption on GNPs/CuFe_2_O_4_ composite.

Pseudo-Second-Order Model		
*q_e_* (mg/g)	*K* (h^−1^)	R^2^
84.46	0.331	0.916

## Supplementary Materials

The following are available online at http://www.mdpi.com/1424-8220/17/6/1292/s1, Figure S1: SEM images of the GNPs/CuFe_2_O_4_ composite, Figure S2: TEM images of the GNPs/CuFe_2_O_4_ composite, Figure S3: Adsorption isotherm for As(III) by GNPs/CuFe_2_O_4_ composite, Table S1: Langmuir and Freundlich isotherm parameters for As(III) adsorption on GNPs/CuFe_2_O_4_ composite, Figure S4: Adsorption of 10 mg GNPs@Fe_2_CuO_4_ composite toward 3 mg/L of As^5+^ and Pb^2+^ for 2 hours, Figure S5: Filter column with a diameter of 2 cm and a height of 10 cm for recyclability.
