# Supplementary material for: Graphene-Supported Spinel CuFe2O4 Composites: Novel Adsorbents for Arsenic Removal in Aqueous Media
Source: Sensors (Basel). 2017 Jun 5;17(6):1292. doi: 10.3390/s17061292 (PMC5492396; doi:10.3390/s17061292)
Supplement: Supplementary file 1 [file sensors-17-01292-s001.pdf]

# Supplementary Materials: Graphene-Supported Spinel $CuFe_2O_4$ Composites: Novel Adsorbents for Arsenic Removal in Aqueous Media

**Duong Duc La, Tuan Anh Nguyen, Lathe A. Jones and Sheshanath V. Bhosale**

This provides further information about the scanning electron microscopy (SEM) and transmission electron microscopy (TEM) images for obtaining the graphene nanoplates (GNPs)/ $CuFe_2O_4$ composite, adsorption isotherm of GNPs/$CuFe_2O_4$ toward As(III), Langmuir and Freundlich isotherm parameters for As (V) adsorption on GNPs/$CuFe_2O_4$ composite, and the assembly of column test. This material is available free of charge via the internet.

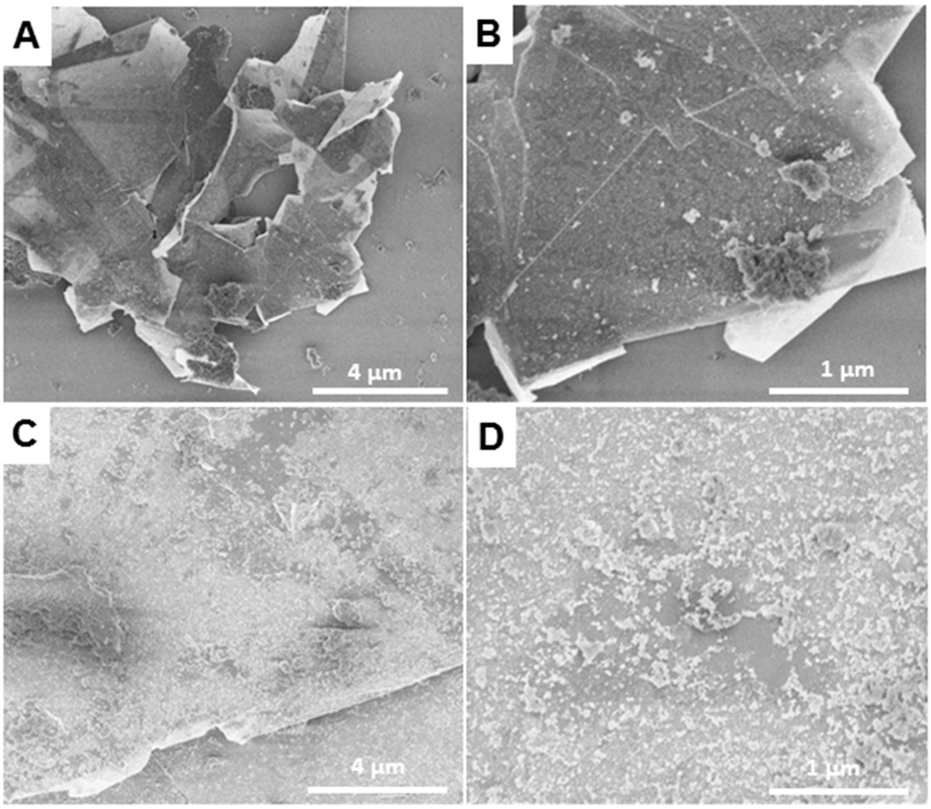


**Figure S1.** (**A**–**D**) low and high resolution SEM images of the GNPs/$CuFe_2O_4$ composite, respectively.

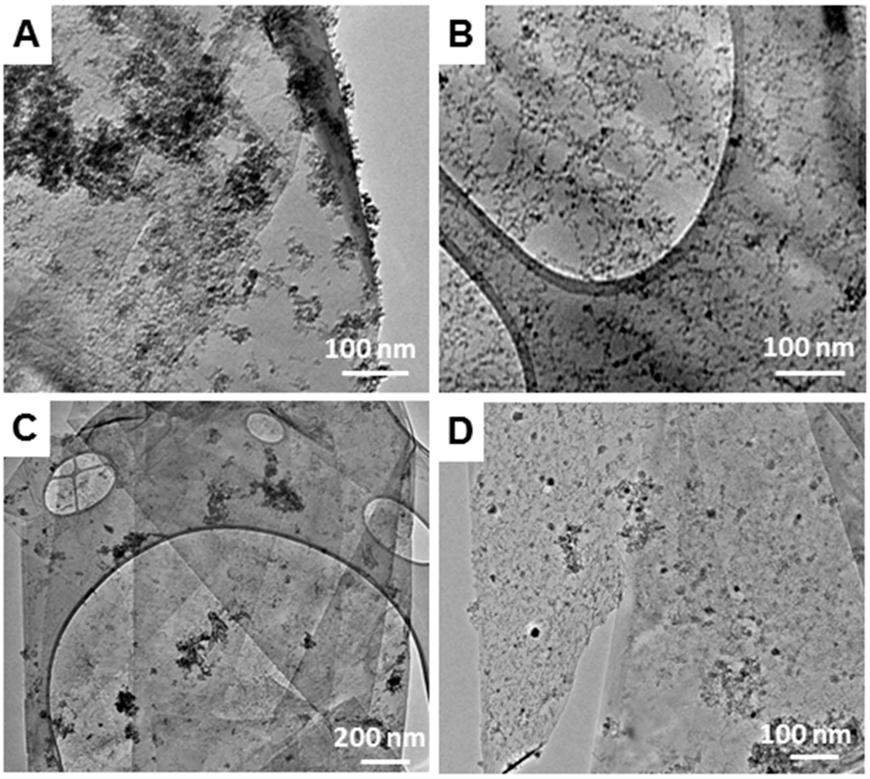


**Figure S2.** (**A**–**D**) low and high resolution TEM images of the GNPs/$CuFe_2O_4$ composite, respectively.

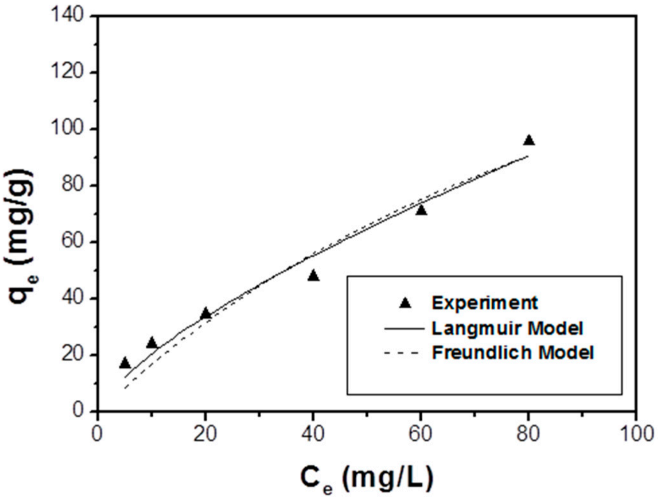


**Figure S3.** Adsorption isotherm for As(III) by GNPs/$CuFe_2O_4$ composite.

**Table S1.** Langmuir and Freundlich isotherm parameters for As(III) adsorption on GNPs/$CuFe_2O_4$ composite.

| | Langmuir Model | | | Freundlich Model | | |
|---|---|---|---|---|---|---|
| | $Q_m$ (mg/g) | $K_L$ (L/mg) | $R^2$ | $K_F$ | n | $R^2$ |
| As(III) | 236.29 | 0.007 | 0.935 | 4 | 0.7 | 0.966 |

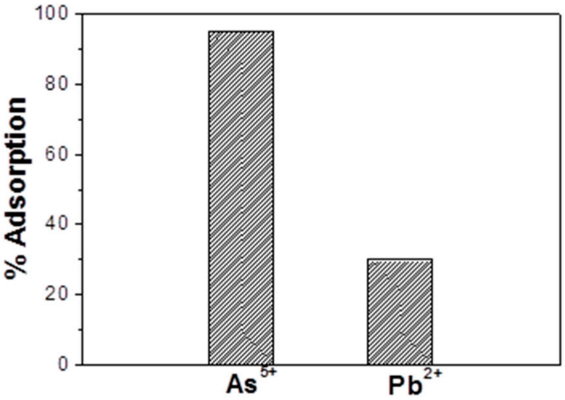


**Figure S4.** Adsorption of 10 mg GNPs@$Fe_2CuO_4$ composite toward 3 mg/L of $As^{5+}$ and $Pb^{2+}$ for 2 h.

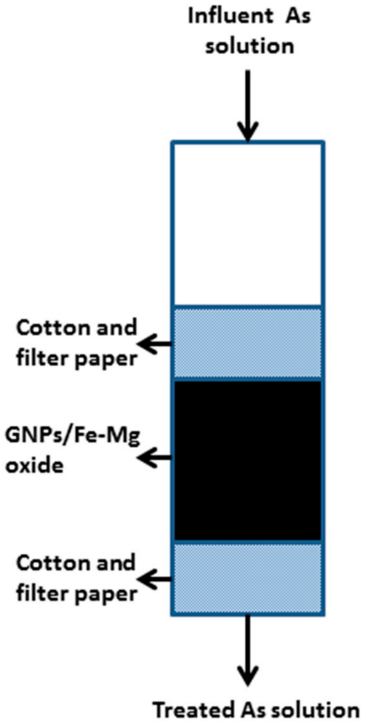


**Figure S5.** Filter column with a diameter of 2 cm and a height of 10 cm for recyclability.
